# Heartbeats and high scores: esports triggers cardiovascular and autonomic stress response

**DOI:** 10.3389/fspor.2024.1380903

**Published:** 2024-04-04

**Authors:** Sascha Ketelhut, Claudio R. Nigg

**Affiliations:** Department of Health Science, Institute of Sport Science, University of Bern, Bern, Switzerland

**Keywords:** esports, mental stress, physical stress, hemodynamics, heart rate variability, energy expenditure

## Abstract

**Introduction:**

Gaming is often labeled as sedentary behavior. However, competitive gaming, also known as esports, involves significant cognitive demands and may induce stress. This study aims to investigate whether the psychophysical demands during esports elicit a physiological stress response.

**Methods:**

Fourteen FIFA 21 and thirteen League of Legends players (23.3 ± 2.8 years) were recruited for the study. Heart rate (HR), root mean square of successive differences between normal heartbeats (RMSSD), peripheral and central blood pressure (BP), pulse wave velocity (PWV), and energy expenditure (EE) were assessed during supine rest, seated rest, and competitive FIFA or League of Legends matches.

**Results:**

No significant group × condition interactions were observed for any of the outcomes. However, there were significant increases in mean HR (*p* < 0.001, $\eta_{p}^{2}$*_ _*= 0.383), RMSSD (*p* = 0.019, $\eta_{p}^{2}$*_ _*= 0.226), peripheral systolic BP (*p* < 0.001, $\eta_{p}^{2}$*_ _*= 0.588), peripheral diastolic BP (*p* = 0.005, $\eta_{p}^{2}$ = 0.272), central systolic BP (*p* = 0.005; $\eta_{p}^{2}$*_ _*= 0.369), central diastolic BP (*p* = 0.016, $\eta_{p}^{2}$*_ _*= 0.313), PWV (*p* = 0.004, $\eta_{p}^{2}$*_ _*= 0.333), and EE (*p* < 0.001, $\eta_{p}^{2}$*_ _*= 0.721) during both games compared to the seated rest condition.

**Conclusion:**

Despite the sedentary nature of esports, the psychophysical demands appear to elicit physiological responses. Interestingly, no significant differences were found between the different game genres.

## 1 Introduction

Esports is an unprecedented cultural phenomenon defined as organized competitive digital gaming played across a spectrum of professionalism, often associated with elements such as spectators, fans, tournaments, leagues, training, skill development, sponsorship, commercial partnerships, and prize money (1). The exponential growth of esports, encompassing both viewership and participation, has raised concerns about the overall well-being of esports athletes (e-athletes) (2, 3). This concern stems from the inherent sedentary nature of video gaming, which has been shown to have detrimental effects on health. Prolonged sitting is associated with an increased risk of developing cardiovascular disease, obesity, and diabetes (4, 5). It is worth noting that sedentary behavior is increasingly recognized as an independent risk factor for adverse health outcomes, regardless of an individual's level of physical activity (4, 6).

Therefore, even though e-athletes may not be physically inactive *per se* (7, 9), extended periods of sedentary gaming could still pose a potential health risk. According to an online survey conducted by Kari et al. (10), e-athletes train for approximately 5.28 h every day throughout the year. Interestingly, most studies on esports define video gaming simply as sitting and primarily focus on potential health concerns related to sedentary behavior. However, it is important to recognize that e-athletes are subjected to intense mental and even physical demands while competing (11, 12). In fact, top athletes in esports can make up to 10 actions per second or 500–600 actions per minute (13). Additionally, they must adaptively cope with the cognitive challenges and emotional stress inherent in competitive settings (14, 16). Potential mental stress experienced during extended and repetitive hours of gaming could increase the risk for chronic stress, which is associated with an elevated risk of cardiovascular diseases as well as mood disorders such as depression (17, 18). This prolonged mental stress, coupled with the sedentary behavior inherent in esports, can contribute to the overall health risk of e-athletes.

The physiological stress responses associated with esports have not been thoroughly investigated. The current literature on physiological responses during video gaming indicates that e-athletes experience notable physiological changes during gameplay (19). Studies have observed increases in heart rate (HR), blood pressure, and energy expenditure (EE) during video gaming compared to rest (19). However, the literature is inconclusive, and variations appear to exist depending on the specific game genres (20) and whether the game setting is casual or competitive (19).

Previous research has shown that higher physical fitness can mitigate cardiovascular reactivity in response to acute psychological stressors (21) and physiological stressors (22). Therefore, it is worth questioning whether physical fitness plays a role in the physiological stress response during gaming and whether promoting physical fitness could serve as an effective preventive measure against gaming-induced stress.

This study aims to assess HR, heart rate variability (HRV), blood pressure, and EE duringesports. Furthermore, it seeks to evaluate whether there are differences in physiological reactions with respect to game genre. Lastly, the study aims to investigate the potential influence of physical fitness and match result on the stress response induced by gaming.

The findings of this study will provide valuable insights into the physical stress experienced by e-athletes and its potential implications for their overall health and well-being. These results can inform future research and the development of interventions aimed at enhancing performance and potentially mitigating health risks in this population.

## 2 Methods

### 2.1 Participants

This observational study included a sample of e-athletes who met specific inclusion criteria: (1) were engaged in esports [either FIFA 21 or League of Legends (LoL)], (2) were between the ages of 16 and 45, (3) had no physical limitations that hindered exercise, (4) provided written informed consent, and (5) were not taking antihypertensive or other cardiovascular medication. Participants were recruited through personal contacts and social media platforms (Facebook, discord, Twitter). The recruitment and data collection period spanned from January 2022 to September 2022.

An *a priori* power analysis was conducted using G*Power (Version 3.1.2; Heinrich Heine Universität, Dusseldorf, Germany). Based on an assumed large effect size (23) and an alpha level of.05, a minimum of 16 participants was deemed necessary to achieve an appropriate power of 0.8. Using these estimated sample sizes, our proposed sample of 27 participants (FIFA = 14, LoL = 13) was more than adequate. Prior to enrollment, the participants were provided with information regarding the study's objectives and procedures, and written consent was obtained from each participant.

### 2.2 Study design

The study took place at the health physiology laboratory of the University of Bern. Participants visited the lab on two separate test days, with a minimum of 48 h between each visit. They were instructed to arrive at the lab at least 4 h after their last meal and to avoid consuming caffeinated or alcoholic beverages, as well as nicotine, for 4 h prior to their visit. Additionally, they were advised not to engage in intense physical activity for at least 24 h before each test day. The experimental procedures of the study were approved by the Ethical Commission of the Faculty of Human Sciences at the University of Bern (Nr. 2021-02-00005).

### 2.3 Procedure

During the first test day, anthropometric parameters and demographic data were collected. Moreover, an incremental exercise test was conducted to determine rate of peak oxygen consumption (VO_2_peak) and peak HR (HRpeak).

**Figure 1:**
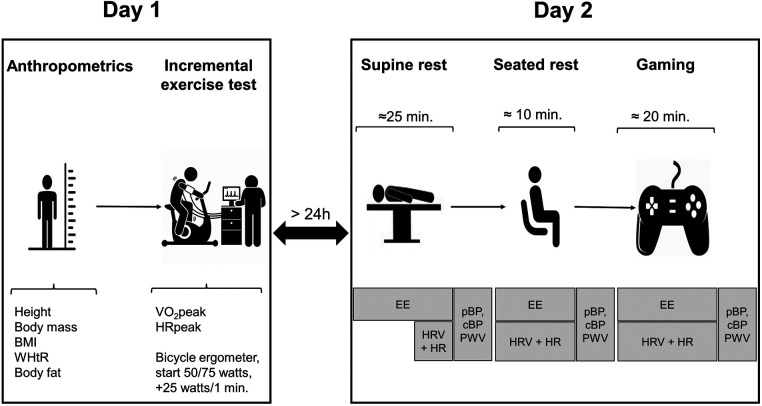
Study design. BMI, body mass index; WHtR, waist-to-height ratio; VO_2_peak, peak oxygen consumption; HRpeak, peak heart rate; EE, energy expenditure; HR, heart rate; HRV, heart rate variability; pBP, peripheral blood pressure; cBP, central blood pressure; PWV, pulse wave velocity.

On the second test day, resting EE was assessed using indirect calorimetry. During the last 5 min of the EE assessment, HR and HRV measurements were obtained. Subsequently, hemodynamic parameters were assessed (Figure 1).

**Figure 2:**
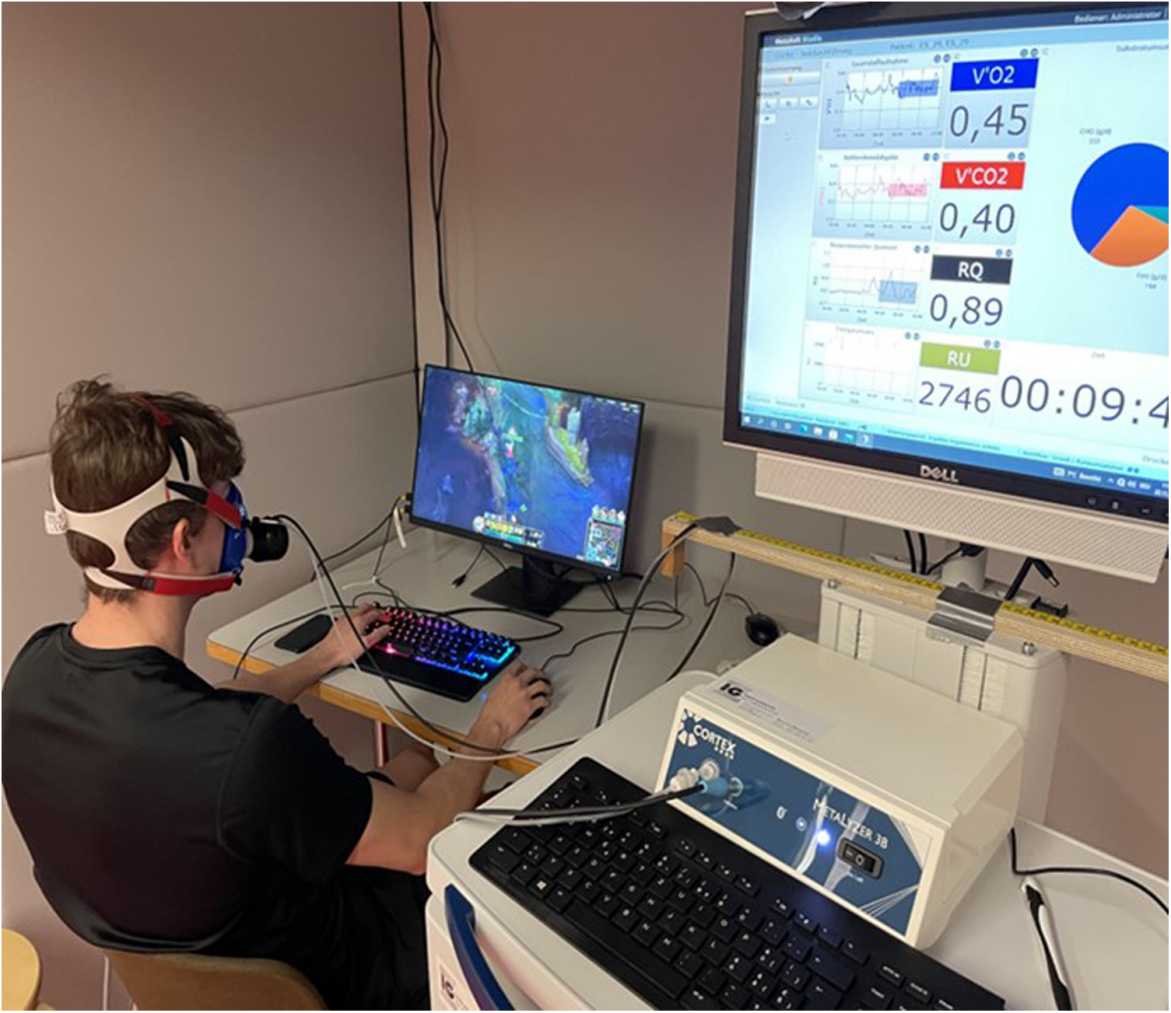
Setup of the gaming session.

Participants then transitioned to a seated position, where EE, HR, and HRV were measured again during a 10-min period. Participants sat on a chair and were instructed to remain calm, recline slightly, and ensure that their feet were flat on the floor, with arms resting on the upper thighs. The use of entertainment media was not allowed. Following this, hemodynamic parameters were recorded. Finally, EE, HR, and HRV were measured during a competitive game of either FIFA 21 or LoL (Figure 2). Hemodynamic parameters were assessed directly after the gaming session (Figure 1). Trained study staff conducted all measurements using standardized procedures and the same equipment. The temperature of the lab was controlled at 20 ± 1°C.

### 2.4 Gaming session

The FIFA e-athletes engaged in a game of FIFA 21 (Electronic Arts, Redwood City, USA) on the PlayStation 4 (Sony, Tokyo, Japan). FIFA 21 is a soccer simulation and the most popular single-player (1 vs. 1) game in the sports genre. The game allows players to control virtual soccer teams and participate in matches. The gameplay involves strategic decision-making, passing, shooting, and defending. Given that FIFA 21 is a single-player game, individual performance is directly linked to match outcomes. While FIFA can be played on PC, it is more commonly played on consoles such as the Xbox and PlayStation, with the gamepad being the most frequently utilized control device. To maintain a competitive environment in the present study, the e-athletes participated in an online seasonal competition called “FIFA 2021 Ultimate Team Champions - Division Rivals”. This competition utilizes a skill-based ranking system to ensure fair matchups. The game was played on a PlayStation 4 (Sony, Tokyo, Japan), where e-athletes either used their own controllers or were provided with a DualShock 4 Wireless Controller (Sony, Tokyo, Japan).

Regarding the LoL e-athletes, they took part in a game of LoL. LoL is a highly popular and complex multiplayer online battle arena (MOBA) video game developed by Riot Games (Riot Games, Los Angeles, USA). In LoL, two teams of five players each compete with the objective of destroying the opposing team's Nexus, a core building located within their base. Each player controls only one character from a bird's-eye view perspective. Players select champions with unique abilities, forming a team composition to complement each other's strengths and exploit the opponent's weaknesses. Due to the team-based nature of the game, individual performance of a player may not necessarily ensure overall team success. The game's intricate mechanics, diverse champions, and dynamic map contribute to a challenging environment that demands a diverse set of skills. LoL is played exclusively on PC using a mouse and keyboard. The athletes in the present study played a match within the ranked tier Solo Queue. This game mode matches athletes with others possessing a similar skill level or rank, thus ensuring balanced gameplay experiences. The game was played on a gaming computer (Mifcom GmbH, Munich, Germany). A gaming mouse (Razer DeathAdder Essential, Razer, California, USA), mousepad, keyboard (Sharkoon Skiller SGK4, Sharkoon Technologies GmbHm, Pohlheim, Germany), and monitor (P2719H, Dell Technologies Inc., Round Rock, USA) was provided or the athletes used their own devices.

Both FIFA 21 and League of Legends (LoL) demand distinct skill sets from their players. Nagorsky and Wiemeyer (24) emphasize the significance of personal attitudes, strategic thinking, and decision-making in LoL. In contrast, FIFA places greater emphasis on competencies such as handling technical issues, adjusting game settings, and reaction time. Due to the team-based nature of LoL, teamwork and receptiveness to team feedback are notably more important in LoL (24).

All e-athletes were instructed to use their primary, personal accounts to ensure they were motivated to win. Additionally, all participants that won their game took part at a raffle where they could win prices. Participants were instructed not to engage in verbal communicate during the gaming session. However, impulsive reactions based on what was happening in the game were permitted and did not need to be deliberately suppressed.

### 2.5 Measurements

#### 2.5.1 Anthropometrics

Height was assessed using a stadiometer. A bioimpedance scale (Tanita RD-545, Tanita Europe BV, Amsterdam, Netherlands) was used to assess weight and calculate body fat. Waist circumference was measured with a precision of 0.1 cm at the midpoint between the iliac crest and the lowest ribs. Body Mass Index (BMI) was calculated as weight in kilograms divided by the square of height in meters (kg/m^2^). The Waist-to-Height Ratio (WHtR) was determined by dividing the waist circumference by the height (waist circumference/height).

#### 2.5.2 Incremental exercise test

An incremental exercise test on a bicycle ergometer (Ergometrics 800s, Ergoline GmbH, Bitz, Germany) was conducted to determine individual HRpeak and VO_2_peak. The test started at 50 or 75 watts (depending on lean body mass and training status) with a stepwise increment of 25 Watts per minute. Participants performed a five-minute warm-up at the respective starting watt level before proceeding to the incremental exercise test. Participants rode on the bicycle until voluntary exhaustion or until a cadence of greater than 60 revolutions per minute could no longer be maintained. Verbal encouragement was provided by the study staff to ensure participants exerted maximal efforts. The test concluded with a 3-min cool-down at 50 watts.

Throughout the test, oxygen consumption was collected and analyzed. The VO_2_peak was calculated as the highest recorded value, using the recorded rolling average of 15-second epochs. HR was monitored throughout the test by a HR monitor using a chest strap.

#### 2.5.3 Cardiac autonomic function

HR and HRV, were measured using a HR monitor and chest strap (Polar RS800 CX®, Polar Electro OY, Kempele, Finland). The RR intervals were recorded at a sampling rate of 1,000 Hz (25). Participants were instructed to breathe normally and refrain from speaking during the measurement. Raw data were processed using the Elite HRV app (Elite HRV Inc., Asheville, United States), which has been validated for its validity and reliability (26). The analysis of HRV included the root mean square of successive differences between normal heartbeats (RMSSD), the standard deviation of all normal-to-normal intervals (SDNN), and HR.

#### 2.5.4 Hemodynamics

Peripheral systolic (pSBP) and diastolic blood pressure (pDBP), as well as central systolic (cSBP) and diastolic blood pressure (cDBP) measurements, along with pulse wave velocity (PWV), were obtained using the Mobil-O-Graph (24 PWA monitor, IEM, Stolberg, Germany). This device is clinically validated for hemodynamic measurements (27). Custom-fit arm cuffs were placed on the participants’ left arm, and at least two readings were obtained for each measurement time point, which were then averaged for analysis.

#### 2.5.5 Ventilation

Ventilation was recorded continuously (breath-by-breath) during the incremental exercise test, the supine rest, the seated rest, and the gaming session using a breath-by-breath gas collection system (Metalyzer 3B, Cortex, Leipzig, Germany). A two-point calibration procedure was conducted according to the manufacturer's guidelines prior to each testing session. The calibration of the oxygen and carbon dioxide sensors was performed with gases of known concentrations. The flow rate was calibrated with a 2-L syringe. In addition, ambient air measurements were conducted before each test.

For the resting EE, participants were instructed to rest in a supine position in a quiet, darkened room, ensuring emotional tranquility. In this position, participants lay flat on their backs with their legs extended and arms resting by their sides, ensuring minimized muscular engagement and facilitating a state of relaxation. The supine rest position was maintained throughout the designated resting period. After a 10-min resting period, values were recorded. EE was then measured for approximately 15 min while participants remained calm and awake. Resting EE was obtained when the participants attained a steady state. Steady states were defined as time intervals of at least 5 min, in which every average minute oxygen consumption and carbon dioxide production changed by less than 10%, and the average respiratory quotient changed by less than 5%.

For the seated and gaming condition, ventilation was assessed throughout the whole condition. The EE for all conditions was calculated from the VO_2_ and VCO_2_ using the Weir equation (28).

### 2.6 Statistical analysis

All statistical analyses were performed using IBM SPSS Statistics v. 27.0 (SPSS, Chicago, IL, USA). The results are presented as means ± standard deviation. Student's *t*-tests were performed to determine the possible differences between FIFA and LoL e-athletes at the baseline. A series of group (LoL vs. FIFA) × condition (seated rest vs. gaming) repeated-measures analysis of variance (ANCOVA) were calculated to compare differences between groups. VO_2_peak and match result (winning or losing) were included as covariates. Significant interactions or main effects were analyzed using a Bonferroni *post hoc* test. Partial eta-squared ($\eta_{p}^{2}$) values were calculated to estimate the effect sizes (small effect: $\eta_{p}^{2}$ = 0.014, medium effect: $\eta_{p}^{2}$ = 0.06, large effect: $\eta_{p}^{2}$ = 0.14) for the interactions. Statistical significance was set at *p *< 0.05.

## 3 Results

No adverse events occurred during the examination sessions in any of the participants. The characteristics of the participants are presented in Table 1. Only one of the LoL e'athlete reported being a semi-professional player earning a share of his main income from esports. All other e-athletes reported being amateur competitive e-athletes, not earning any substantial income from competing. Only male e-athletes participated in this study. There were no significant differences in age, BMI, WHtR, body fat, sitting hours, and gaming hours between the groups (Table 2). According to BMI, 10 e-athletes were classified as being overweight (5 FIFA, 5 LoL) and one (LoL) as being obese. Regarding WHtR only 4 e-athletes (1 FIFA, 3 LoL) were above the established 0.5 health risk threshold for WHtR (29). Similarly, 4 e-athletes (1 FIFA, 3 LoL) had values above the obesity thresholds with respect to body fat (30). FIFA e-athletes reported significantly higher engagement in sports compared to LoL e-athletes and reached a significantly higher VO_2_peak. According to VO_2_max cutoff values (31) 8 e-athletes (6 FIFA, 2 LoL) were classified as superior to excellent, 16 e-athletes (8 FIFA, 8 LoL) were classified as good to fair, and 3 e-athletes (3 LoL) were classified as very poor to poor. The gaming sessions were higher in the LoL compared to the FIFA group (1217 ± 180 s vs. 864 ± 55 s).

**Table 1:** Participants’ outcomes at supine rest.

Outcome	Total (*n* = 27)	FIFA (*n* = 14)	LoL (*n* = 13)	*p*-values	Cohens *d*
HRmean (min^−1^)	67 ± 14	67 ± 15	67 ± 13	.913	.043
RMSSD (ms)	63 ± 39	54 ± 30	73 ± 46	.202	.505
SDNN (ms)	85 ± 32	81 ± 29	88 ± 37	.621	.193
pSBP (mmHg)	122 ± 8	119 ± 5	124 ± 10	.082	.697
pDBP (mmHg)	72 ± 8	71 ± 9	72 ± 7	.655	.174
cSBP (mmHg)	125 ± 12	123 ± 10	127 ± 14	.439	.386
cDBP (mmHg)	75 ± 6	73 ± 5	76 ± 6.7	.406	.416
PWV (m/s)	5.2 ± 0.3	5.1 ± 0.3	5.2 ± 0.3	.326	.386
EE (kcal/day)	2,051 ± 218	2,008 ± 191	2,098 ± 243	.293	.414

Data expressed as the mean ± standard deviations. *p*-values indicate the differences between FIFA and LoL players. HRmean, mean heart rate; RMSSD, root mean square of successive differences between normal heartbeats; SDNN, standard deviation of all normal-to-normal intervals; pSBP, peripheral systolic blood pressure; pDBP, peripheral diastolic blood pressure; cSBP, central systolic blood pressure; cDBP, central diastolic blood pressure; PWV, pulse wave velocity; EE, energy expenditure.

**Table 2:** Participants’ characteristics.

Outcome	Total (*n *= 27)	FIFA (*n *= 14)	LoL (*n *= 13)	*p*-values	Cohens *d*
Age (years)	23 ± 3	24 ± 3	23 ± 3	.348	.368
Height (m)	1.79 ± 0.05	1.79 ± 0.05	1.79 ± 0.05	.858	.070
Weight (kg)	76.4 ± 10.0	76.8 ± 8.1	76.0 ± 12.1	.842	.078
BMI (kg/m^2^)	24 ± 3	24 ± 2	24 ± 3	.861	0.68
WHtR	0.45 ± 0.04	0.45 ± 0.03	0.45 ± 0.04	.983	.008
Body fat (%)	18.2 ± 5.7	17.0 ± 4.7	19.6 ± 6.5	.240	.464
Sitting hours (h/day)	6.8 ± 2.5	7.4 ± 2.4	5.6 ± 2.4	.130	.215
Sports engagement (h/week)	6.0 ± 3.5	7.7 ± 3.3	4.1 ± 2.7	.008	1.163
Gaming hours (h/week)	9.7 ± 5.9	8.4 ± 4.9	12.3 ± 7.6	.168	.664
VO_2_mpeak (ml/min/kg)	47.4 ± 9.1	50.8 ± 9.1	43.7 ± 8.0	.044	.818

Data expressed as the mean ± standard deviations. *p*-values indicate the differences between FIFA and LoL players. BMI, body mass index; WHtR, waist-to-height ratio; VO_2_peak, peak oxygen consumption.

Regarding the outcomes at baseline, no significant differences could be detected between the groups (Table 1). According to reference values (32), one LoL e'athlete had high normal blood pressure, and one was classified as hypertensive.

No significant condition × group interactions could be detected for any of the outcomes (Table 3). However, significant main effects for time could be detected for mean HR [F(1,23) = 14.254, *p* < 0.001, $\eta_{p}^{2}$ = 0.383]; RMSSD [F(1,22) = 6.428, *p* = 0.019; $\eta_{p}^{2}$ = 0.226], pSBP [F(1,25) = 35.620, *p* < 0.001, $\eta_{p}^{2}$ = 0.588], pDBP [F(1,25) = 9.356, *p* = 0.005, $\eta_{p}^{2}$ = 0.272], cSBP [F(1,25) = 10.471, *p* = 0.005, $\eta_{p}^{2}$ = 0.369], cDBP [F(1,25) = 7.288, *p* = 0.005, $\eta_{p}^{2}$ = 0.313], PWV [F(1,21) = 0.520, *p* = 0.004, $\eta_{p}^{2}$ = 0.333], and EE [F(1,25) = 64.659., *p* < 0.001, $\eta_{p}^{2}$ = 0.721]. No significant main effect for group could be detected in any of the outcomes. VO_2_peak and match result had no effect on the group × condition interactions or the main effect for time or group (all *p*_s_ > 0.05).

**Table 3:** Changes in outcomes between seated rest and gaming conditions for FIFA and LoL players.

Outcome	FIFA (*n *= 14)		LOL (*n *= 13)		*p*-values		
	Seated rest	Gaming	Seated rest	Gaming	Time × group	Time	Group
HRmean (min^−1^)	69 ± 15	77 ± 18	74 ± 11	81 ± 17	.590	<.001	.443
RMSSD (ms)	69 ± 41	51 ± 29	51 ± 24	48 ± 21	.120	.019	.367
SDNN (ms)	102 ± 44	84 ± 34	83 ± 36	81 ± 33	.231	.131	.432
pSBP (mmHg)	126 ± 14	141 ± 14	125 ± 12	141 ± 12	.827	<.001	.884
pDBP (mmHg)	80 ± 11	86 ± 10	81 ± 8	88 ± 14	.799	.005	.736
cSBP (mmHg)	137 ± 14	138 ± 16	122 ± 13	138 ± 11	.100	.005	.337
cDBP (mmHg)	87 ± 8	93 ± 8	85 ± 7	92 ± 11	.772	.016	.675
PWV (m/s)	5.2 ± 0.4	5.5 ± 0.6	5.1 ± 0.4	5.6 ± 0.4	.479	.004	.792
EE (kcal/min)	1.63 ± 0.19	2.06 ± 0.31	1.51 ± 0.19	1.86 ± 0.20	.404	<.001	.149

Data expressed as the mean ± standard deviations. HRmean, mean heart rate; RMSSD, root mean square of successive differences between normal heartbeats; SDNN, standard deviation of all normal-to-normal intervals; pSBP, peripheral systolic blood pressure; pDBP, peripheral diastolic blood pressure; cSBP, central systolic blood pressure; cDBP, central diastolic blood pressure; PWV, pulse wave velocity; EE, energy expenditure.

## 4 Discussion

The present study aimed to investigate the physiological stress responses of e-athletes during competitive gaming sessions and explore the potential influence of physical fitness. The results of this study show that gaming results in significant physiological reactions, which are likely due to psychophysical stress during gaming and may have implications for the overall health and well-being of e-athletes.

Regarding the outcomes at baseline, no significant differences were detected between the FIFA and LoL group. Interestingly, FIFA e-athletes reported significantly higher engagement in sports compared to LoL e-athletes and achieved a significantly higher VO_2_peak, indicating better aerobic fitness. It is possible that e-athletes who compete in sports genre video games may exhibit a greater inclination toward physical sports (9). This assumption is supported by a study on virtual football players revealing high levels of physical activity, with 87% meeting the World Health Organization recommendations for physical activity (33). These levels of physical activity are much higher than those reported in other studies of e-athletes from different game genres (7).

wAnalyzing the group × condition interactions, no significant differences were found for any of the outcomes, indicating that the physiological stress responses during gaming sessions did not differ significantly between the FIFA and LoL group. However, significant main effects for time were observed for HR, RMSSD, pSBP, pDBP, cSBP, cDBP, PWV, and EE suggesting that the gaming sessions elicit physiological responses.

### 4.1 Cardiac autonomic function

During the gaming sessions, the mean HR showed a significant increase, reaching 40 ± 11% of the HRpeak. The highest HR recorded during the gaming session corresponds to 53 ± 0.1% of HRpeak. These results are consistent with previous research conducted by Yeo et al. (34), which reported a similar increase in HR during a game of LoL. However, conflicting results have been reported for other game titles (35, 36). Regarding the game of FIFA, studies conducted by Siervo et al. (20) and Zimmer et al. (37) reported no significant increase in HR during the game. Interestingly, Siervo et al. (38) even reported a decrease in heart rate while playing FIFA.

The differences in HR responses among studies might be attributed to the competitive or casual nature of the gaming environment. This is support by a study from Chaput et al. (39), who intentionally stimulated a competitive environment in their study and found a significant increase in HR during video gaming compared to a seated control condition. Adachi & Willoughby (40), assessed HR during different game titles and found that only highly competitive games resulted in an increase in HR, further corroborating the influence of competitiveness on HR response during gaming. Our results and these previous findings suggest that differences in HR response are more related to the competitive character of the game than the specific game title.

Regarding HRV, significant main effects for time were observed for RMSSD, reflecting a reduced vagal activity of the heart. However, no effects on SDNN were evident. In a study assessing a game of LoL, Yeo et al. (34), reported a significant increase in low-frequency power, a significant decrease in high-frequency power, and an increase in the ratio between low- and high-frequency components of HRV, indicating a shift towards sympathetic dominance and reduced parasympathetic activity during gameplay. These results align with Chaput et al. (39), who also observed an increase in the low- to high-frequency components ratio of HRV during gaming compared to resting conditions. Due to the limited physical demand of the video games assessed (FIFA and LoL), the results suggest that esports induces mental stress, which has been reported to trigger sympathetic activation and parasympathetic withdrawal (41, 42). Interestingly, no effect of the match result on HRV parameters was detected. This contrasts with a recent study by Machado et al. (43), which indicated that the outcome of the game affected perceived stress and HRV parameters after gaming. However, it is worth noting that in this study, these parameters were assessed after the game rather than throughout.

### 4.2 Hemodynamics

Playing video games resulted in a significant increase in peripheral and central blood pressure, as well as PWV. Again, no differences could be detected between the two game genres. The results are in line with Chaput et al. (39), who reported a significant increase in systolic as well as diastolic blood pressure during a competitive video game play compared to resting values. Siervo et al. (20) found a significant increase only in pDBP during 1 h of playing violent video games. In a more recent study the same author (38) reported significant higher pSBP during video gaming compared to watching TV. However, the significant differences resulted from a reduction while TV viewing and no changes while video gaming. Similar to our study, the authors found no difference between game genres. Conversely, two studies found differences between game genres, showing that violent video games resulted in more pronounced increases in blood pressure (44, 45). Previous studies suggest that the violent content within video games may create an internal aggressive state that increases arousal and triggers cardiovascular stress responses (45, 46). However, this is not directly supported by the results of our study, as LoL and FIFA are not considered violent video games.

Regrettably, prior investigations have not examined central blood pressure or PWV. PWV, which characterizes the velocity of the central pulse wave and indicates arterial stiffness, serves as an early indicator for existing structural vascular changes and subsequent cardiovascular risks (47). Evidence suggests that PWV is more strongly associated with preclinical organ damage and is a better predictor of future cardiovascular events than peripheral blood pressure (48). The central blood pressure reflects the afterload of the heart and correlates with the myocardial oxygen consumption. Accordingly, the prognostic significance of central blood pressure is evaluated higher than that of peripheral blood pressure (49).

The increase in hemodynamic parameters during gaming could possibly be explained by an sympathetic α-adrenergic stimulation that induces an activation of endothelial cells and smooth muscle cells resulting in systemic vasoconstriction, and increased total peripheral resistance (50, 51).

### 4.3 Energy expenditure

The EE significantly increased duringesports. During the gaming session the e-athletes reached metabolic equivalent of task (MET) values of 1.6 ± 0.3, which is equivalent to low intensity exercise (52). However, when considering the MET values based on the individual resting EE rather than the standard 3.5 references from the literature, the mean MET values were found to be 1.4 ± 0.2, falling within the definition of sedentary behavior. The highest VO_2_ values reached by the e-athletes were 9.64 ± 2.56 ml/min/kg corresponding to 21 ± 0.1% of their individual VO_2_peak.

In a recent study by Kocak (53), MET values during gaming were reported to reach 1.9 MET, indicating light physical activity. On the other hand, Mansoubi et al. (54) found that playing video games resulted in 1.4 METs. While Chaput et al. (39), reported a significant increase in EE during video gaming compared to resting condition, Zimmer et al. (37), did not observe significant changes in EE and VO_2_ in participants playing FIFA or the first-person multiplayer shooter Counter-Strike: Global Offensive. Similarly, Haupt et al. (55) did not detect an increase in EE, though it's worth noting that both studies did not employ a competitive setting.

While video gaming does involve repetitive hand and finger movements to control the gamepad, mouse, and keyboard, the impact on EE from these actions may be relatively minor. Instead, the mental stress induced by cognitive engagement and emotional responses seems to trigger sympathetic activation, leading to an increase in EE through beta-adrenergic mechanisms (56). Once again, the competitive setting may provide an explanation for the conflicting results.

### 4.4 Practical implications

The study provides valuable insights into the physiological stress responses during esports. The findings reveal that gaming sessions, characterized by prolonged sitting and intense mental demands, elicit physiological changes, including increased HR, blood pressure, and EE and decreases in HRV. Considering these responses is crucial when addressing the health risks associated with esports, as gamers may be at risk of experiencing chronic stress outcomes. While short episodes of stress may not pose a significant health risk, repeated and prolonged cardiovascular activation can lead to vascular hypertrophy, progressively increasing peripheral resistance and contributing to the development of established hypertension (57, 58).

This becomes particularly relevant as prolonged gaming sessions become more common (59). Notably, the average online video gamer in America plays for 6.44 h per week (60), and pro-gamers, in particular, spend approximately 9.4 h per day playing video games (61). While casual gaming may not induce strong physiological reactions, competitive gaming settings seem to pose a potential problem. As esports tournaments often span several hours, and online leagues continue to expand, gamers may find themselves increasingly exposed to extended competitive gaming sessions.

Thus, coaches and e-athletes should carefully consider how to optimize training regimens and implement appropriate recovery strategies to reduce stress and promote overall well-being (62). Apart from recovery and stress management strategies, physical exercise may represent a potential countermeasure. Although the present results show that physical fitness had no effect on the stress response experienced during gaming, it is possible that acute exercise may attenuate the stress response. Studies have shown that acute exercise can not only reduce the response to physical stress (22, 63) but also mitigate the response to mental stress (64). Consequently, incorporating physical exercise into the training regimen and conducting short exercise sessions before esports sessions could present a promising approach to decrease acute stress response and mitigate the long-term effects of prolonged and repeated periods of high stress, thus helping to prevent negative health outcomes.

### 4.5 Limitations

While this study offers valuable insights into the physiological stress responses of e-athletes, some limitations should be considered. One limitation is that the study employed a cross-sectional design, which only provides a snapshot of the participants’ physiological stress responses during gaming sessions. Longitudinal studies would be beneficial to investigate the long-term effects of gaming on the health and well-being of e-athletes. Second, we only took blood pressure measurements at the end of the gaming session. Continuous monitoring would have provided a more comprehensive view of the stress response over time. However, as participants’ need for full control over their arms and hands continuous monitoring was not feasible during gaming. Third, the study did not account for potential variations in performance levels within and between the two groups, which may have influenced the observed stress responses. A study by Poulus et al. (16) suggests an association between esports performance level and levels of mental toughness, indicating that individuals who are more successful have higher levels of mental toughness. Fourth, the total duration of the gaming sessions differed between the groups, potentially influencing the results. Fifth, the study assessed physiological reactions during competitive online environments but did not examine the stress response in official tournament settings. Physiological reactions in official tournaments might differ due to increased pressure. Nevertheless, the competitive online environment could provide a more accurate representation of the situations e-athletes encounter in their daily gaming experiences. Lastly, only male participants took part in this study, despite gender not being a criterion for inclusion or exclusion. This gender bias is common in esports research (65).

## 5 Conclusion

This study sheds light on the physiological stress responses of e-athletes during gaming sessions and their potential impact on overall health. Gaming sessions, characterized by prolonged sitting and intense mental demands, lead to significant physiological changes, including increased HR, blood pressure, EE and reduced HRV. Game genre, match results, and physical fitness level had no effect on the stress response. These findings highlight the importance of addressing the health risks associated with esports, as prolonged cardiovascular activation may have adverse effects. Coaches and e-athletes should optimize training regimens to reduce stress responses and ensure a healthy gaming environment without compromising game specific performance. Future research should continue to investigate various game titles and discern the mechanisms (mental, cognitive) behind the psychological responses.

## Data Availability

The raw data supporting the conclusions of this article will be made available by the authors, without undue reservation.
